# Effects of L-Arginine Supplementation during Late Gestation on Reproductive Performance, Piglet Uniformity, Blood Profiles, and Milk Composition in High Prolific Sows

**DOI:** 10.3390/ani10081313

**Published:** 2020-07-30

**Authors:** Jinsu Hong, Lin Hu Fang, Jae Hark Jeong, Yoo Yong Kim

**Affiliations:** 1 Department of Agricultural Biotechnology and Research Institute of Agriculture and Life Science, Seoul National University, Seoul 08826, Korea; jinsu.hong@sdstate.edu (J.H.); john1988123@hotmail.com (L.H.F.); jaehark323@hanmail.net (J.H.J.); 2 Department of Animal Science, South Dakota State University, Brookings, SD 57007, USA

**Keywords:** arginine, late gestation, sow, reproductive performance, piglet uniformity

## Abstract

**Simple Summary:**

Arginine is one of the functional amino acids that enhances the growth of fetus and placenta development. Since the fetal growth and the nutrient requirement for fetuses are increased hugely during the late gestation period in high-prolific sows, supplementation of L-arginine could have a positive influence on the reproductive performance of sows and piglet uniformity. In the present study, increasing inclusion level of L-arginine linearly increased alive litter weight at birth and litter weight gain during lactation. However, the piglet uniformity at birth decreased linearly, as dietary arginine level increased in the late gestation period. We concluded that the inclusion level of arginine in the diet for late gestating sows, by up to 1.5%, could improve the alive litter weight and weight gain of their progeny. The piglet uniformity at birth was decreased due to the increase of survival for piglets with light birth weight.

**Abstract:**

This study was conducted to evaluate the effects of L-arginine supplementation levels during late gestation on reproductive performance and piglet uniformity in high prolific sows. A total of 60 F1 multiparous sows (Yorkshire × Landrace), with an average body weight of 238.2 kg, were allotted to one of three treatment groups in a completely randomized design. The dietary treatments were divided by the supplementation level of arginine during the late-gestation period, from day 70 to farrowing, as follows—(1) CON: corn-soybean meal-based basal diet (Arg 0.72%), (2) Arg10: basal diet + L-Arg 0.28% (Arg 1.0%), and (3) Arg15: basal diet + L-Arg 0.79% (Arg 1.5%). The same lactation diet was provided ad libitum to sows during the lactation period. There were no significant differences in body weight and backfat thickness in sows during late-gestation and lactation. Dietary arginine levels had no significant influences on the number of total born, stillbirth, and born alive. However, increasing inclusion level of L-arginine supplementation tended to increase (*p* < 0.10) alive litter weight linearly, and also linearly increased (*p* < 0.05) the piglet weight gain and litter weight gain during the lactation period. In piglet uniformity, the standard deviation of piglet birth weight (*p* < 0.05) and the coefficient of variation for piglet birth weight (*p* < 0.10) increased linearly, as dietary arginine levels increased in the late gestation period. Increasing L-arginine supplementation to late gestating sows linearly increased (*p* < 0.05) the blood concentrations of arginine and ornithine at day 90 and day 110 of gestation. On the other hand, dietary arginine levels in late gestation did not affect the blood parameters related to the nitrogen utilization. Increasing dietary arginine levels for the late gestating sows did not affect the milk composition for colostrum and milk at day 21 of lactation. In conclusion, the inclusion level of arginine in the diet for late gestating sows, by up to 1.5%, could improve the alive litter weight at birth and litter weight gain during lactation, whereas the piglet uniformity at birth was decreased due to the increase of survival for fetuses with light birth weight.

## 1. Introduction

The high prolific sows are developed to improve the number of piglets and sow productivity in the swine industry [1]. However, as they have greater litter size, some problems were reported that the proportion of small piglets at birth, within-litter variation of piglet birth weight, and mortality of suckling piglets were also increased [2,3,4]. These problems are caused by several maternal factors, such as inadequate nutrient intake of late gestation, high number of fetus, insufficient cervical space, or insufficient reproductive tract for fetus development [5,6,7]. Therefore, various studies were conducted to minimize these problems and improve the piglet uniformity of high prolific sows with nutritional strategies including sow body condition, nutrients type or ratio, and functional amino acids [8,9,10].

Arginine is one of the functional amino acids that signals embryonic and fetal development [11], and it was reported that arginine could partly be attributed to improving piglet birth weight and the uniformity of piglets at birth [12,13]. Dietary arginine intake was shown to increase the synthesis of nitric oxide (NO) and polyamines [14,15]. The nitric oxide thus produced resulted in increased blood flow to the placenta [16,17] and improved delivery of essential nutrients from maternal to fetal blood [18]. Polyamines also showed positive effects on embryogenesis and placental growth [19]. Thus, arginine was shown to influence placental growth and fetal development via the above mechanism [20,21].

Fetal growth is rapid during the late gestation period and nutrient requirement for the fetus also increases greatly [22]. The low birth weight of piglets was reported to increase the proportion of stillborn piglets considered to be at a greater risk of mortality and morbidity [23,24]. Since supplying adequate nutrient to sows during the late gestation period is important, an increased feeding method was suggested in order to meet their nutrient requirement. Although increased feeding during late gestation is likely to meet the nutrient requirement of sows and improve piglet birth weight [25,26], it also showed a negative effect on sow body condition and postpartum agalactia [27,28], resulting in poor milk production and lactation feed intake [29,30]. With the effects of arginine, if the nutrient delivery efficiency from the dam to the fetus in late gestation increased by additional arginine supplementation, it could partly be attributed to increase in the birth weight of small fetuses and piglet uniformity at birth.

Most of the previous studies for evaluating the effect of arginine in gestating sows were investigated in the early-gestation period [31,32,33,34] or whole gestation period [12,13,35,36]. Additionally, most previous studies for the effect of arginine supplementation in sows, investigated the effect of 1% arginine supplementation in sow diet. Thus, there is a need to investigate the effects of dietary arginine below 1% of supplementation level keeping in mind the market cost of L-arginine, in order to apply this in the field.

Therefore, we hypothesized that dietary supplementation with L-arginine in late gestation might provide more nutrients and oxygen from the maternal to fetus tissue, for fetal development and survival, thereby enhancing the reproductive performance in the sows. The objective of this study was to evaluate the effects of arginine supplementation levels in late gestation on reproductive performance, piglet uniformity, blood profiles, and milk composition in high prolific sows.

## 2. Materials and Methods

All experimental procedures involving animals were conducted in accordance with the Animal Experimental Guidelines provided by the Seoul National University Institutional Animal Care and Use Committee (SNU-160819-9).

### 2.1. Animals

A total of 60 F1 multiparous sows (Yorkshire × Landrace) with an average body weight (BW) of 238.2 kg, average backfat thickness (BFT) of 20.4 mm, and an average parity of 4.9, were allotted to one of three treatments based on the BW, BFT, and parity in a completely randomized design, when the sows reached day 70 of gestation. All sows had undergone two artificial inseminations, according to the estrous cycle after weaning, and pregnancy was checked at day 35 of gestation, using an ultrasound scanner. Before starting the experiment, the second parity sows were fed a 2.2 kg/day gestation diet, and sows of over third parity were fed a 2.4 kg/day gestation diet.

### 2.2. Experimental Diet

The dietary treatment had different levels of arginine content in gestation diet—Arg 0.72% (CON): corn-soybean meal (SBM)-based diet with L-Arg 0% and L-Ala 1.63%; Arg1.0% (ARG10): corn-SBM-based diet with L-Arg 0.28% and L-Ala 1.01%; and Arg1.5% (ARG15): corn-SBM based diet with L-Arg 0.79% and L-Ala 0%. The dietary arginine content during late gestation (70–110 days) was as follows; Arg0.72%: 15.8–17.3 g/day, Arg1.0%: 22–24 g/day, and Arg1.5%: 33–36 g/day. Alanine was chosen for the isonitrogenous control because Ala is not toxic and not a substrate for Arg synthesis, but Ala is extensively catabolized by pigs [12]. The experimental diet contained 3265 kcal of metabolizable energy (ME)/kg, 13.55% crude protein (CP), 0.74% total lysine, 0.23% total methionine, 0.45% total threonine, and 0.11% total tryptophan. The lactation diet contained 3265 kcal of ME/kg; 13.68% crude protein. The L-Arginine and L-Alanine (Ajinomoto Co. Inc., Tokyo, Japan) were supplemented in the experimental diets in the dry form. The calcium and total phosphorus of the experimental diets met the nutrient requirements of NRC [37], and other nutrients met or exceeded the nutrient requirements of the NRC [38]. The analyzed and calculated chemical composition of the experimental diets are presented in Table 1.

### 2.3. Animal Management

All sows were fed 2.2 kg/day (second parity) or 2.4 kg/day (over third parity) of the experimental diet, once a day (08:00), according to their parity, and the feed was gradually reduced to 0.2 kg/day for 5 days before the date of farrowing. After farrowing, sows were fed a lactation diet of 1 to 5 kg/day during the first 5 days postpartum, and then they were fed ad libitum until weaning.

All sows were accommodated in individual gestation stalls (2.20 × 0.64 m), where the indoor temperature was regulated at an average of 20 °C by an automatic ventilation system. At day 110 of gestation, the sows were moved from the gestation barn to the farrowing crates (2.50 × 1.80 m), after washing and disinfecting their body, especially the mammary gland and vulva. All sows were allowed to farrow without induction agents, and they were given assistance for dystocia as needed. The room temperature of the lactating barn was kept at 28 ± 2 °C, and the farrowing crate under a heating lamp was kept at 32 ± 2 °C. Air conditioning in the lactating barn was regulated automatically by the ventilation/air-conditioner system. After weaning, the sows were moved to the breeding barn for the next estrous cycle.

After farrowing, the piglets were cross-fostered within the treatment group until 24 h postpartum to balance the suckling intensity of sows, with equalization of litter size, and thus to minimize any effect of the initial litter size potentially affecting the litter growth. Tail docking, Fe-dextran 150 ppm (Gleptosil^®^, Alstoe Ltd., Sheriff Hutton, UK) injection, and castration (for male piglets) were performed on all piglets, 3 days after birth. All piglets suckled milk only from the sow, and creep feed was not provided until weaning.

#### 2.3.1. Body Weight, Backfat Thickness, and Lactation Feed Intake

The BW and BFT of sows were measured at day 70 and 110 of gestation, at 24 h postpartum, and at day 21 of lactation. The BW of sows was measured by an electric scale (CAS Co. Ltd., Yangju-si, Gyeonggi-do, Korea) for sows, and BFT was measured at the P_2_ position (mean value from both sides of the last rib and 65 mm away from the backbone) using an ultrasound device (Lean Meter^®^, Renco Corp., Minneapolis, MN, USA). The daily feed wastage was recorded during lactation and the lactation feed intake was measured when measuring BW and BFT of lactating sows, at day 21 of lactation.

#### 2.3.2. Reproductive Performance

After farrowing, the numbers of total born piglets, stillbirth, mummy, and alive piglets were recorded, and the BW of alive piglets, stillborn, and mummy were measured by an electric scale (CAS Co. Ltd., Yangju-si, Gyeonggi-do, Korea). When measuring the BW of piglets, ear notching was performed for the experiment. Then, cross-fostering the piglets within the same treatment group was done within 12 h postpartum, to equalize litter size among the sows. The number of piglets and their BW were measured at day 21 of lactation for calculating litter weight, piglet weight, and their weight gain. Farrowing time was recorded from the start of the farrowing to the total release of the placenta. After the farrowing, the whole placenta was collected in a bucket and the weight was measured immediately. The placenta weight was divided by litter weight to calculate the placental efficiency. The helping frequency during farrowing for sows with dystocia was recorded.

#### 2.3.3. Piglet Uniformity

The coefficient of variation (CV) and standard deviation (SD) were calculated from each weight of alive piglet at 24 h postpartum and day 21 of lactation. Additionally, the distributions of piglet BW at birth and day 21 of lactation were measured.

#### 2.3.4. Blood Profiles

Blood collection from sows (*n* = 8 for each treatment) was taken by venipuncture of the jugular vein, using 10 mL disposable syringes at day 70, 90, and 110 of gestation, 24 h postpartum, and at day 21 of lactation. All blood samples were enclosed in serum tubes (SST^TM^II Advance, BD Vacutainer, Becton Dickinson, Plymouth, UK), as well as ethylenediamine tertaacetic acid (EDTA) tubes (BD Vacutainer K_2_E, Becton Dickinson, Plymouth, UK), and centrifuged at 1957× *g* and 4 °C for 15 min (5810R, Eppendorf, Hamburg, Germany), after clotting at room temperature for 30 min. The upper liquid (serum) of the blood was separated to a microtube (Axygen, Union City, CA, USA) and stored at −20 °C in a freezer, until later analysis. Blood urea nitrogen (BUN; kinetic UV assay, Roche, Mannheim, Germany), total protein (colorimetry, Roche, Mannheim, Germany), creatinine (kinetic colorimetry assay, Roche, Mannheim, Germany), and urea (kinetic UV assay, Roche, Mannheim, Germany) were analyzed by Modular Analytics (Hitachi Ltd., Tokyo, Japan). Plasma amino acid was analyzed by LC–MS/MS (3200 QTRAP, AB SCIEX, Framingham, MA, USA).

#### 2.3.5. Milk Composition

Colostrum samples (*n* = 8 for each treatment) were taken from functional mammary glands at 24 h postpartum, and milk samples (*n* = 8 for each treatment) were taken at day 21 of lactation. Colostrum and milk were collected in 50 mL conical tubes (SPL Life Sciences Co., Ltd., Pocheon-si, Gyeonggi-do, Korea) from the first and second teats, after an intravascular injection with 5 IU oxytocin (Komi oxytocin inj., Komipharm International Co., Ltd., Siheung-si, Gyeonggi-do, Korea) in the ear. After collection, the samples were stored in a −20 °C freezer until further analysis. The contents of casein, fat, protein, lactose, total solid, and solid not fat in colostrum and milk were determined using a Milkoscan FT 120 (FOSS, Hillerød, Denmark).

#### 2.3.6. Statistical Analysis

All collected data were analyzed by least squares mean comparisons and were evaluated with the general linear model (GLM) procedure of SAS (SAS Institute Inc., Cary, NC, USA). Orthogonal polynomial contrasts were used to determine linear and quadratic effects, by increasing the arginine supplementation level. The individual sow was used as the experimental unit in growth performance, reproductive performance, blood profiles, and milk composition. Their litter was used as the experimental unit in piglet growth and piglet uniformity. The model included the effects of the treatment group and sampling date, as well as interactions of the treatment group and sampling date for blood parameters. The piglet BW distribution within litter on day 0 and day 21 of lactation was analyzed by the FREQ procedure of SAS (SAS Institute, Cary, NC, USA). The CHISQ option was used to assess chi-square tests of homogeneity or independence and measures of association among categorical variables, which helps to identify the statistically significant difference in the frequency of piglet BW in litters among the three treatment groups of sows. The differences were declared to be significant at *p* < 0.05, and the determination of tendency for all analyses was *p* ≥ 0.05 and *p* < 0.10.

## 3. Results

The arginine supplementation levels in the late-gestating sows’ diet did not affect body weight, backfat thickness, and the lactation feed intake of sows (Table 2).

In reproductive performance, dietary arginine levels during late gestation had no influence on the number of piglets for total born, stillborn, mummy, and born alive (Table 3). However, piglet birth weight showed a quadratic response (*p* < 0.03) such that the piglet birth weight was decreased by the arginine levels from 0.72% to 1.0%, whereas it was increased by the arginine level from 1.0% to 1.5%. Additionally, increasing the inclusion level of dietary arginine tended to increase (*p* < 0.10) the alive litter weight linearly. Increasing the dietary arginine levels in late gestation did not affect the farrowing time, helping frequency, and placenta weight.

Increasing dietary arginine levels in late gestation linearly increased (*p* < 0.05) the litter weight at day 21, and the litter weight gain, such that litter weight and litter weight gain for ARG15 tended to be greater than those for CON (Table 4). The piglet weight at day 21 of lactation was linearly increased (*p* < 0.05) as the dietary arginine level increased in late gestation. Additionally, increasing the dietary arginine level in late gestation linearly increased (*p* < 0.05) piglet weight gain, such that the piglet weight gain for ARG15 tended to be greater than that for CON.

**Figure 1:**
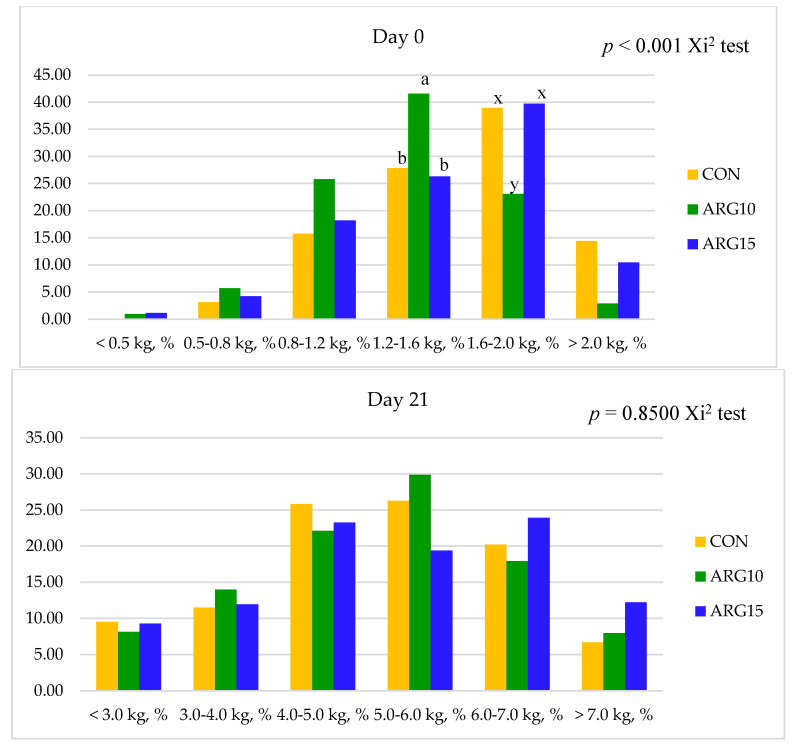
Effect of arginine supplementation level in late gestating sows on piglet distribution into body weight classes on day 0 and day 21 of lactation by the FREQ procedure, with Chi-square test. CON: corn-SBM-based diet with Arg 0.72%, Arg10: corn-SBM-based diet with Arg 1.0%, Arg15: corn-SBM-based diet with Arg1.5%.

The effect of dietary arginine levels in late gestation on the piglet uniformity is presented in Table 5. The SD for piglet birth weight was linearly increased (*p* < 0.05), and the CV for piglet birth weight had a tendency of linear increase (*p* < 0.10) as the dietary arginine level increased in late gestation. The piglet BW distribution at birth showed a significant difference (*p* < 0.01) among dietary treatment (Figure 1). However, dietary arginine levels in late gestation did not affect the piglet uniformity and BW distribution at day 21 of lactation.

The blood concentration of AAs in the gestating sows is presented in Table 6. Increasing arginine supplementation to late-gestating sows linearly increased (*p* < 0.05) the blood concentrations of arginine and ornithine at day 90 and day 110 of gestation, respectively. On the other hand, the blood concentrations of alanine at day 110 of gestation for sows fed a diet with higher dietary arginine were linearly decreased (*p* < 0.03) compared to those for sows fed the isonitrogenous control diet. Additionally, glutamine concentration for sows at day 110 of gestation was quadratically increased (*p* < 0.05), as the dietary arginine level increased. There were no significant differences in blood concentrations of other AA, among dietary treatments. The plasma concentrations of arginine, lysine, and methionine for day 90 of gestation were greater (*p* < 0.05) than those for day 70 of gestation. The plasma concentrations of alanine and glutamine for day 110 of gestation were greater (*p* < 0.05) than those for day 70 of gestation, whereas the plasma concentrations of citrulline, glycine, leucine, ornithine, taurine, and valine for day 110 of gestation were less (*p* < 0.05) than those for day 70 of gestation. There were interactions (*p* < 0.05) between arginine and the date effect in the plasma concentration for ornithine such that there was no significant difference in the plasma ornithine concentration for day 70 of gestation, whereas plasma ornithine concentrations at day 90 of gestation for ARG10 and ARG15 treatments were greater (*p* < 0.05) than that for CON treatment, and plasma ornithine concentration at day 110 of gestation for ARG15 treatment was greater (*p* < 0.05) than those for CON and ARG10 treatments.

The dietary arginine levels for the late-gestating sows had no significant influence on the blood concentrations of BUN, creatinine, total protein, and urea for sows in the late gestation period (Table 7). The blood concentrations of BUN, creatinine, and urea for day 110 of gestation were greater (*p* < 0.05) than those for day 70 of gestation. There were no interactions between arginine and the date effect in the blood concentrations for BUN, creatinine, total protein, and urea. Increasing the dietary arginine levels for sows during late gestation did not affect the milk composition of colostrum and milk (day 21 of lactation) in lactating sows (Table 8).

## 4. Discussion

Supplementing sow diet with L-arginine during the late gestation did not influence the gain of BW and BF in sows for the late gestation period and loss of BW and BF in sows for the lactation period. This was in agreement with the result of Quesnel et al. [39], who reported that dietary supplementation with 25.5 g/day L-arginine from day 77 to farrowing did not affect the BW after farrowing and BF before farrowing of sows. Additionally, Bass et al. [40] reported that dietary supplementation with 1% L-arginine for gestating sows from day 93 to day 110 had no influence on BW loss, following farrowing or during lactation. However, they observed that improved late gestation BW gain of sows fed a diet with 1% L-arginine was revealed in parity 0 and parity 1, whereas, there was no difference in late gestation BW gain compared to the control animals and 1% L-arginine-supplemented sows in parity 2+, implying that supplementation with 1% L-arginine partially met the arginine requirement in gilts or sows with parity 1 sows, not in sows with parity 2+. The difference between the current study and that of Bass et al. [40] with regard to the effect of dietary arginine in late gestation on the changes of BW or BF for sows could have been due to the difference in the parities of sows in the treatment group. Additionally, the lack of difference in the changes of BW and BF for sows during the whole experiment period could partly have been due to the same nitrogen content of diets among the dietary treatments, with the addition of L-Alanine as the isonitrogenous control.

Dietary effects of L-arginine was reported such that supplementation of L-arginine was found to increase nitric oxide, enhancing the delivery of essential nutrients from maternal to fetal blood [16,17,18], and increasing the polyamines necessary for embryogenesis and placental growth [14,15,20]. Since the nitric oxide and polyamines are important for angiogenesis and embryogenesis, arginine enhanced the growth of fetus and placenta development [19,20]. The litter size, including the number of total born or born alive was not affected by the dietary arginine effect in the present study, which was in agreement with the results from the studies of Quesnel et al. [39] and Nantapaitoon et al. [41], who reported that 1% L-arginine supplementation for late-gestating sows had no influence on the number of piglets at birth. However, previous studies reported that arginine supplementation enhanced conceptus survival or sow litter size in the period of early-gestation [33,42], or whole gestation [12,13,36]. The number of developing embryos is decided in early gestation, because most of the embryonic losses occurred during early gestation [43], and the dead embryos were not reabsorbed by the uterus after day 40 of gestation [44]. Thus, the differences among studies with regard to the effects of dietary arginine on the litter size of sows could have been due to differences in the supplemented period for the diet with L-arginine.

In the present study, we observed the tendency of a linear increase in alive litter weight, due to increasing arginine levels in the diet from 0.72% to 1.5%, which was in agreement with the result from the studies of Liu et al. [45], who reported that addition of 1% L-arginine in the diet for late-gestating sows increased alive litter birth weight compared to that of the control diet. Similarly, Wu et al. [46] reported a significant increase in average BW of total piglet born alive due to an addition of 1% L-arginine for gestating sows from day 90 to farrowing. Additionally, Che et al. [13] reported that dietary L-arginine supplementation from day 30 to day 110 of gestation had greater total litter weight and live litter weight than those for sows fed a diet with arginine from day 30 to day 90 of gestation. The increased alive litter weight due to dietary L-arginine could be explained by the increased arginine concentration in blood (Table 6) and numerical increase in placenta efficiency (Table 3). In general, a well-developed placenta is associated with enhanced fetal growth, whereas impaired placental growth is related to the intra-uterine growth retardation (IUGR). Additionally, Wu and Meininger [47] reported that dietary arginine improved the blood flow between the placenta and fetus, which affected nutrients and oxygen transfer to the fetuses. Thus, additional L-arginine supplementation specifically during late gestation was likely to improve placental growth and fetal development via the above mechanism, resulting in a linear increase in the total litter weight at birth.

The placenta is the pivotal organ that is responsible for the supply of nutrients and biologically active substances from the maternal to the fetal system. Placenta vascular functions are vital for providing maternal nutrients or oxygen, and blood flow to the fetuses related to the fetal growth [48,49]. Nitric oxide (NO), produced from arginine via nitric oxide synthase, is involved in the vasodilation of the maternal systemic circulation, regulation of uterine, and fetoplacental blood flow [14,50,51]. Gao et al. [36] reported that 1% L-arginine supplementation from day 22 to day 114 of gestation increased the placental weight for all live-born piglets. Liu et al. [45] reported that 1% L-arginine supplementation during late gestation of sows improved efficiency in the utilization of dietary nutrients through the expression of microRNAs, and gene expressions of VEGFA (vascular endothelial growth factor A) and eNOS (endothelial nitric oxide synthase), in the umbilical vein. Additionally, Wu et al. [46] reported that 1% L-arginine supplementation for sows in late gestation increased VEGFA and eNOS gene expression in placental surface vessels. In this context, supplementation of additional L-arginine enhanced the nutrients and oxygen transfer to the fetuses via the umbilical cord [41,47]. In the present study, dietary arginine level had no influence on the placenta weight and placental efficiency, which was in agreement with the result from the study of Garbossa et al. [34], who reported that supplementing 1% L-arginine from day 25 to day 53 of gestation did not affect placental efficiency; and Bass et al. [40] who reported that supplementing 1% L-arginine from day 93 of gestation to farrowing did not affect the placenta weight. Thus, the similarities between the current study and those of Garbossa et al. [34] and Bass et al. [40] with regards to the effects of dietary arginine on placenta weight and placental efficiency, could be attributed to the fact that the effect of increasing dietary arginine was not enough to show significant differences in the placenta weight and placental efficiency.

To investigate the carry-over effect of arginine supplementation in late gestation on the sows and their offspring, we measured the weight of piglets and litters from day 0 to day 21 of lactation. In the present study, the litter weight and piglet weight after cross-fostering for ARG10 treatment were lower than other treatments, which was because the piglets were cross-fostered within the same treatment group, based on the litter size of each sow. As a result, the number of piglets for each treatment was similar, but the litter weight and piglet weight had some differences among the treatments. Interestingly, despite the different initial weight of piglets or litters, increasing the level of dietary arginine in the late gestation period linearly increased the litter weight gain and piglet weight gain from day 0 to day 21 of lactation. This was in contrast to the results from the studies of Quesnel et al. [39], Bass et al. [40], and Nuntapaitoon et al. [41], who reported that 1% of L-arginine supplementation for late gestating sows had no significant influences on the piglet weight, piglet weight gain, and litter growth rate during the lactation period. The differences between the current study and those of other studies with regard to the effects of dietary arginine on the litter weight gain and piglet weight gain could have been due to the differences in the parity of sows and the period of arginine supplementation. This is because the average parity of the sows used in the current study was higher than those of other studies (4.9 vs. 3.0), and the period of arginine supplementation (45 days vs. 22–38 days) was longer than other studies. Litter weight gain is correlated with milk production and nutrient concentrations in milk [52,53]. Additionally, sow nutrition during late gestation is important for the colostrum yield of sows, and nutrient intake and sow body condition are important for the milk yield of sows [54]. Though there were no differences in the lactation daily feed intake, the losses of BW and BF, and milk composition, the litter weight gain and piglet weight gain was improved due to increasing dietary arginine levels during the late gestation period, implying that arginine supplementation for late-gestating sows could partly contribute to an increase of milk production due to the increased the mammary plasma flow and the efficiency of milk production in lactating sows [55]. On the other hand, previous studies showed that dietary L-arginine supplementation in gestating sows increased concentrations of nutrients, IGF-1, and IgG in the colostrum [41,56]. When dietary arginine was supplemented in the sow diet during the late gestation period, the plasma concentration of the growth hormone in the umbilical venous of sows and the blood oxygen saturation of neonatal piglets were increased compared to those of pigs fed the diet without L-arginine supplementation [41,45]. In the current study, these factors could partly contribute positively to the piglet growth in the lactation period.

The piglet uniformity at birth, known as within-litter birth weight variation, was negatively correlated to piglet survival and postnatal growth, resulting in economic losses to the swine producer [8,9]. An increasing level of dietary arginine in late gestating sows decreased the uniformity of piglet at birth in the current study. This result was in contrast with the result from the study of Quesnel et al. [39], who reported that the CV in piglet birth weight was decreased due to 1% of arginine supplementation from day 77 to farrowing. However, several studies reported that additional arginine supplementation had no significant effect on the piglet uniformity at birth in gilts, from day 30 to 114 of gestation [12], and in gestating sows from day 25 to day 53 [34], day 30 to day 90 or day 114 [13], and from day 85 to farrowing [41]. It should be noted that arginine supplementation in the diet for gestating sows increased the placental blood flow and thereby enhanced fetal development. In the present study, the percentage of light weight piglets (less than 800 g BW) from sows fed the diet with L-arginine (6.6% and 6.4%) was higher than twice that from sows fed the control diet (3.1%). This suggests that arginine supplementation in the sow diet during late gestation increased the placental blood flow for the small fetuses and thereby enhanced their birth weight and survival rate. Additionally, Nuntapaitoon et al. [41] reported that arginine supplementation enhanced the number of piglets with heavier birth weight (>1.35 kg) and reduced the proportion of growth-restricted piglets. Regarding the result of alive litter weight and proportion of piglet birth weight in the current study, dietary arginine supplementation increased the placental–fetal blood flow, which might allow transferring more nutrients and oxygen to the fetuses from the smallest one to the biggest one. However, the growth-retarded pig fetus might have a limitation of fetal growth due to the placental function, as small placenta has slower blood flow, lower uptake of essential amino acids, and fewer and less dense areolae [24,57,58]. Thus, the reduced piglet uniformity at birth could partly be attributed to the increase of placental blood flow for the fetuses due to arginine supplementation, which enhanced the weight of the fetus from the lightest to the heaviest one.

The plasma concentration of arginine and ornithine as dietary arginine level increase was in agreement with the results from the studies of Gao et al. [36], who reported that dietary supplementation with 1% of arginine increased plasma ornithine concentrations at day 90 of gestation, and Che et al. [13], who reported that increasing level of dietary arginine supplementation increased plasma ornithine concentrations at day 90 and day 110 of gestation. Additionally, the interaction between dietary arginine levels and blood sampling dates for ornithine appeared in the current study such that the blood ornithine concentration increased with time as the arginine supplementation level increased. Arginine is the precursor for the synthesis of many biological molecules including polyamines, ornithine, proline, glutamine, NO, and protein [14,59]. Classically arginine is converted to ornithine by arginase, and it is subsequently converted to polyamines, proline, glutamate, and glutamine. Ornithine derived from arginine is an important precursor for the synthesis of polyamines and proline in maternal and conceptus tissue [60]. Proline plays a crucial role in polyamine synthesis in both the porcine placenta and fetal intestine [61]. Therefore, the increase in plasma concentration for arginine due to dietary arginine supplementation could have resulted in an increase of plasma ornithine concentration. Increasing the dietary arginine levels, quadratically increased the glutamine concentration for sows at day 110 of gestation in the current study. Gao et al. [36] reported that dietary supplementation with 1% arginine from day 22 to day 114 of gestation did not affect the glutamine concentrations in the plasma on day 40, 70, and 90 of gestation in sows. Glutamine is an important amino acid to carry ammonia from peripheral tissues to the kidney, where the amide nitrogen is hydrolyzed by the enzyme glutaminase. In the kidney, glutamine is hydrolyzed by the enzyme glutaminase to produce glutamate and ammonium ion (NH_4_^+^), which is excreted in the urine. Furthermore, the metabolic wastes including ammonia and β-hydroxybutyrate were reduced when arginine was supplemented in late gestation [62], which implied that the pig fetus might efficiently utilize dietary amino acids [13]. On the other hand, glutamine and arginine metabolism is closely related to several pathways, including immuno-inflammatory response and antioxidant status. Coëffier and Dechelotte [63] reported that combined administration of arginine and glutamine resulted in synergistic effects on the inflammatory response, but arginine reduced glutamine protection against oxidative stress. However, there is a lack of information on the effect of supplementing arginine on glutamine metabolism, in terms of late gestating sows. Therefore, in the present study, which pathways of glutamine associated with the arginine metabolism was affected significantly by dietary arginine levels in the late gestating sows was unclear. The linear reduction of alanine concentration of sows on day 110 of gestation in the current study could be attributed to the fact that alanine was used as an isonitrogenous source in the experimental diets.

Increasing the level of dietary arginine in the diets for the late gestating sows had no significant influences on the blood components associated with protein utilization, such as BUN, creatinine, total protein, and urea concentration in the late gestating sows. Arginine supplementation in the gestating sows increased the circulating levels of arginine and its metabolites, resulting in an enhancement of protein synthesis in the fetus [11,59]. Moreover, the requirement of the arginine-family of amino acids was increased, especially in the critical period of the embryo/fetal development such as the late gestation period in sows [15,60,64]. Bass et al. [40] reported that supplementation of 1% arginine during the final 3 weeks of gestation did not affect the BUN of sows. Gao et al. [36] reported that 1% arginine supplementation during day 22 to 114 of gestation had no effect on the urea concentration in blood of sows. Additionally, Che et al. [13] reported that supplementation of 1% arginine during the gestation period from day 30 to day 90 or day 110 for sows had no significant influence on the concentration of total protein on day 90 and day 110 of gestation. With regards to previous studies, although additional arginine supplementation stimulated vasodilation, blood flow, and subsequent improvement in nutrients and oxygen transfer from the sows to the fetus, the effect of dietary arginine levels in the current study was not enough to have significant differences in the blood components involved in protein utilization of late gestating sows.

Dietary arginine supplementation in the diet for late gestating sows did not affect the composition of colostrum and milk. Nuntapaitoon et al. [41] reported that arginine supplementation in the sow diet during late gestation had no impact on colostrum and milk yield. Additionally, Krogh et al. [56] reported that arginine supplementation did not affect milk production in multi-parous sows. The quality and quantity of milk in early lactation were more dependent on the body reserves of sows, and they were more dependent on both dietary intake and body reserves. In the current study, there were no significant differences in the BW and BFT of sows during the whole experimental period and the lactational feed intake. Thus, increasing arginine supplementation during late gestation had no influence on the composition of colostrum and milk at day 21 of lactation.

## 5. Conclusions

In conclusion, increasing the level of dietary L-arginine supplementation in late gestating sows tended to linearly increase the alive litter weight, and linearly increased litter weight gain and piglet weight gain during the lactation period. However, the piglet uniformity at birth was linearly decreased by an increase in dietary arginine level from 0.72% to 1.5% due to the increased number of piglets with light birth weight of less than 0.8 kg of BW. Therefore, dietary L-arginine could be supplemented in late-gestating sow’s diet at 1.5% to improve alive litter weight, survival rate of neonates with light birth weight, and piglet growth.

## Figures and Tables

**Table 1:** The diet formulation and chemical composition of the experimental diet.

Item	Gestation Diets		
	CON ^1^	ARG10	ARG15
**Ingredients, %**			
Corn	75.16	75.38	76.02
Soybean meal	12.57	12.57	12.26
Wheat bran	1.64	1.93	2.35
Palm kernel meal	3.00	3.00	3.00
Tallow	2.48	2.32	2.07
L-lysine HCl (78%)	0.26	0.26	0.26
DL-methionine (99%)	0.05	0.04	0.04
L-arginine (99%)	0.00	0.28	0.79
L-alanine (98%)	1.63	1.01	0.00
MDCP	1.46	1.46	1.46
Limestone	1.15	1.15	1.15
Vit. mix ^2^	0.10	0.10	0.10
Min. mix ^3^	0.10	0.10	0.10
Choline chloride-50	0.10	0.10	0.10
Salt	0.30	0.30	0.30
Total	100.00	100.00	100.00
**Chemical Composition**			
Dry matter, % ^4^	90.59	90.66	90.88
Crude protein, % ^4^	13.44	13.46	13.36
Ether extract ^4^	6.72	6.38	5.68
Crude ash, % ^4^	3.58	3.64	3.78
ME, kcal/kg ^5^	3265.03	3265.07	3265.03
Total lysine, % ^5^	0.74	0.74	0.74
Total methionine, % ^5^	0.24	0.23	0.23
Total threonine, % ^5^	0.45	0.45	0.45
Total tryptophan, % ^5^	0.11	0.11	0.11
Total arginine, % ^5^	0.72	1.00	1.50
Calcium, % ^5^	0.75	0.75	0.75
Total phosphorus, % ^5^	0.60	0.60	0.60

^1^ CON: corn-soybean meal (SBM)-based diet with Arg 0.72%, Arg10: corn-SBM-based diet with Arg 1.0%, Arg15: corn-SBM-based diet with Arg1.5%. ^2^ Provided per kg of diet: Vitamin A, 8000 IU; Vitamin D_3_, 1600 IU; Vitamin E, 32 IU; d-biotin, 64 g; Riboflavin, 3.2 mg; Calcium pantothenic acid, 8 mg; Niacin, 16 mg; Vitamin B_12_, 12 μg; and vitamin K, 2.4 mg. ^3^ Provided per kg of diet: Se, 0.1 mg; I, 0.3 mg; Mn, 24.8 mg; CuSO_4_, 54.1 mg; Fe, 127.3 mg; Zn, 84.7 mg; and Co, 0.3 mg. ^4^ Analyzed value. ^5^ Calculated value.

**Table 2:** Effects of arginine supplementation levels on body weight and back-fat thickness in late-gestating sows.

	Treatment ^1^			SEM ^2^	*p*-Value		
	CON	ARG10	ARG15		Diet	Lin.	Quad.
**Body Weight, kg**							
Day 70	237.1	239.1	238.4	3.58	0.98	0.90	0.85
Day 110	258.2	256.3	252.7	3.33	0.79	0.50	0.99
BW gain (70–110 days)	21.1	17.3	14.3	2.46	0.53	0.27	0.79
24 h postpartum	233.7	230.8	227.9	3.73	0.82	0.54	0.92
Day 21 of lactation	229.4	224.9	218.5	3.97	0.53	0.26	0.95
BW loss (0–21 days)	−4.3	−5.9	−9.4	1.67	0.44	0.20	0.96
**Backfat Thickness, mm**							
Day 70	20.7	21.4	19.1	0.77	0.49	0.35	0.46
Day 110	22.4	22.6	20.7	0.84	0.59	0.37	0.66
BF gain (70–110 days)	2.1	1.3	1.6	0.3	0.51	0.57	0.30
24 h postpartum	21.0	21.1	20.3	0.85	0.91	0.72	0.84
Day 21 of lactation	20.1	19.8	18.6	0.79	0.71	0.43	0.90
BF loss (0–21 days)	−0.9	−1.3	−1.7	0.38	0.69	0.40	0.85
Lactation feed intake, kg/day	4.82	4.95	4.97	0.114	0.85	0.62	0.77

^1^ CON: corn-SBM-based diet with Arg 0.72%, Arg10: corn-SBM-based diet with Arg 1.0%, Arg15: corn-SBM-based diet with Arg1.5%. ^2^ Standard error of the mean. Lin.: linear, Quad.: quadratic, BW: body weight, BF: backfat.

**Table 3:** Effects of arginine supplementation levels on reproductive performance in late-gestating sows.

	Treatment ^1^			SEM ^2^	*p*-Value		
	CON	ARG10	ARG15		Diet	Lin.	Quad.
**No. of Pigs**							
Total born	13.7	15.0	14.8	0.50	0.53	0.46	0.41
Stillborn	0.7	0.7	0.8	0.12	0.93	0.73	0.89
Mummy	0.2	0.3	0.1	0.08	0.49	0.31	0.53
Born alive	12.8	14.0	13.9	0.45	0.35	0.31	0.31
Total litter weight, kg	19.61	19.91	21.49	0.521	0.30	0.13	0.73
Alive litter weight, kg	18.47	19.01	20.69	0.506	0.19	0.07	0.81
Piglet birth weight, kg	1.57 ^A^	1.38 ^B^	1.52 ^A^	0.036	0.08	0.85	0.03
Farrowing time, min	182.4	186.2	205.7	9.33	0.58	0.31	0.82
Helping frequency	0.80	0.81	1.00	0.141	0.82	0.55	0.85
Placenta weight, kg	3.74	3.74	3.90	0.224	0.95	0.76	0.90
Placenta efficiency	5.80	5.89	6.02	0.246	0.94	0.73	0.98

^1^ CON: corn-SBM-based diet with Arg 0.72%, Arg10: corn-SBM-based diet with Arg 1.0%, Arg15: corn-SBM-based diet with Arg1.5%. ^2^ Standard error of the mean. ^A,B^ Means in the same row with different superscript letters were significantly different (*p* < 0.10). Lin.: linear, Quad.: quadratic.

**Table 4:** Effects of arginine supplementation levels on litter performance in late-gestating sows.

	Treatment ^1^			SEM ^2^	*p*-Value		
	CON	ARG10	ARG15		Diet	Lin.	Quad.
**No. of Piglets**							
After-fostering	11.5	11.6	11.6	0.10	0.90	0.77	0.72
Day 21 of lactation	10.7	10.4	10.9	0.16	0.52	0.49	0.36
**Litter Weight, kg**							
After-fostering	17.67 ^a^	16.01 ^b^	17.73 ^a^	0.326	0.04	0.63	0.01
Day 21 of lactation	53.74 ^B^	53.31 ^B^	60.27 ^A^	1.332	0.05	0.02	0.32
Weight gain (0–21 days)	36.06 ^B^	37.17 ^AB^	42.58 ^A^	1.262	0.07	0.02	0.64
**Piglet Weight, kg**							
After-fostering	1.55 ^a^	1.38 ^b^	1.53 ^a^	0.031	0.04	0.82	0.01
Day 21 of lactation	5.03	5.14	5.51	0.103	0.13	0.04	0.78
Weight gain (0–21 days)	3.48 ^B^	3.74 ^AB^	3.98 ^A^	0.093	0.08	0.03	0.66

^1^ CON: corn-SBM-based diet with Arg 0.72%, Arg10: corn-SBM-based diet with Arg 1.0%, Arg15: corn-SBM-based diet with Arg1.5%. ^2^ Standard error of the mean. ^a,b^ Means in the same row with different superscript letters were significantly different (*p* < 0.05). ^A,B^ Means in the same row with different superscript letters were significantly different (*p* < 0.10). Lin.: linear, Quad.: quadratic.

**Table 5:** Effects of arginine supplementation levels on piglet uniformity in late-gestating sows.

	Treatment ^1^			SEM ^2^	*p*-Value		
	CON	ARG10	ARG15		Diet	Lin.	Quad.
**Piglet Uniformity at Birth**							
Avg. BW, kg	1.57	1.38	1.52	-	-	-	-
SD	274.0	293.5	334.3	12.72	0.13	0.04	0.94
CV	18.4	21.8	22.9	1.01	0.15	0.07	0.41
**Piglet Distribution into Birth Weight Classes**							
<0.5 kg, %	0	1.0	1.2	0.30	0.24	0.15	0.38
0.5–0.8 kg, %	3.1	5.6	4.2	0.79	0.44	0.59	0.25
0.8–1.2 kg, %	15.8	25.8	18.2	2.36	0.21	0.87	0.07
1.2–1.6 kg, %	27.9 ^b^	41.5 ^a^	26.3 ^b^	4.39	0.03	0.49	0.01
1.6–2.0 kg, %	39.0 ^ab^	23.0 ^b^	39.7 ^a^	5.55	0.08	0.64	0.03
>2.0 kg, %	14.5	2.9	10.4	5.67	0.15	0.70	0.06
**Piglet Uniformity at day 21**							
Avg. BW, kg	5.03	5.14	5.51	-	-	-	-
SD	1186.8	1198.1	1277.4	47.64	0.69	0.41	0.84
CV	24.6	24.4	25.1	1.17	0.97	0.85	0.89
**Piglet Distribution into BW Classes at day 21**							
<3 kg, %	9.5	8.1	9.3	1.41	0.91	0.99	0.67
3–4 kg, %	11.5	14.0	11.9	1.46	0.77	0.99	0.48
4–5 kg, %	25.8	22.1	23.3	2.39	0.83	0.72	0.62
5–6 kg, %	26.3	29.9	19.4	2.17	0.14	0.12	0.19
6–7 kg, %	20.2	17.9	23.9	2.01	0.48	0.36	0.42
>7 kg, %	6.7	8.0	12.2	1.80	0.41	0.19	0.86

^1^ CON: corn-SBM-based diet with Arg 0.72%, Arg10: corn-SBM-based diet with Arg 1.0%, Arg15: corn-SBM-based diet with Arg1.5%. ^2^ Standard error of the mean. ^a,b^ Means in the same row with different superscript letters were significantly different (*p* < 0.05). Lin.: linear, Quad.: quadratic, Avg. BW: average body weight, SD: standard deviation, CV: coefficient of variation.

**Table 6:** Effects of arginine supplementation levels on plasma amino acid profile in late-gestating sows.

	Treatment ^1^			SEM ^2^	*p*-Value					
	CON	ARG10	ARG15		Diet	Lin.	Quad.	Group	Date	Group × Date
**Alanine, μmol/L**										
Day 70	609.5	487.0	454.0	46.75	0.49	0.27	0.56	0.09	<0.01	0.51
Day 90	709.1	710.5	652.0	37.24	0.79	0.53	0.79			
Day 110	878.4 ^A^	916.3 ^A^	628.7 ^B^	53.05	0.06	0.03	0.26			
**Arginine, μmol/L**										
Day 70	154.5	255.0	195.8	23.65	0.31	0.66	0.14	<0.01	<0.01	0.27
Day 90	262.9 ^b^	349.1 ^b^	482.7 ^a^	29.44	<0.01	<0.01	0.88			
Day 110	196.4 ^b^	301.2 ^a^	366.9 ^a^	24.34	<0.01	<0.01	0.34			
**Aspartic Acid, μmol/L**										
Day 70	14.0	14.3	13.5	1.33	0.97	0.88	0.89	0.69	0.15	0.74
Day 90	18.8	31.4	18.3	4.79	0.46	0.82	0.23			
Day 110	14.4	14.0	14.4	0.87	0.98	0.96	0.85			
**Citrulline, μmol/L**										
Day 70	128.0	109.3	114.5	5.64	0.54	0.48	0.34	0.96	<0.01	0.58
Day 90	114.8	133.8	127.3	6.99	0.54	0.58	0.35			
Day 110	97.8	89.7	98.9	4.19	0.68	0.80	0.40			
**Cystine, μmol/L**										
Day 70	2.00	0.33	1.00	0.408	0.38	0.47	0.20	0.70	0.50	0.75
Day 90	0.88	1.50	1.29	0.308	0.71	0.68	0.49			
Day 110	3.40	2.33	1.00	0.928	0.57	0.31	0.93			
**Glutamic Acid, μmol/L**										
Day 70	240.5	260.3	198.0	30.72	0.72	0.58	0.66	0.60	0.07	0.81
Day 90	173.3	211.6	189.3	16.13	0.63	0.81	0.36			
Day 110	170.6	153.8	152.6	13.82	0.84	0.64	0.76			
**Glutamine, μmol/L**										
Day 70	260.5	220.7	249.5	23.86	0.84	0.95	0.58	0.27	0.01	0.48
Day 90	343.8	328.9	289.4	14.64	0.32	0.14	0.88			
Day 110	299.8 ^a^	330.8 ^a^	259.9 ^b^	10.39	0.03	0.04	0.04			
**Glycine, μmol/L**										
Day 70	1224.5	1186.7	1073.5	38.59	0.27	0.13	0.85	0.58	0.03	0.76
Day 90	1206.0	1291.5	1204.9	30.62	0.43	0.83	0.20			
Day 110	1070.9	1105.3	1117.9	39.67	0.88	0.66	0.85			
**Histidine, μmol/L**										
Day 70	93.0	103.3	93.0	5.31	0.72	0.90	0.46	0.36	0.21	0.98
Day 90	103.9	116.5	96.6	6.80	0.51	0.56	0.31			
Day 110	90.8	97.0	88.7	3.60	0.69	0.71	0.42			
**Isoleucine, μmol/L**										
Day 70	107.0	121.3	104.0	6.11	0.51	0.72	0.32	0.65	0.81	0.92
Day 90	120.4	125.8	115.7	3.46	0.52	0.50	0.35			
Day 110	134.2	119.5	109.4	11.82	0.69	0.42	0.84			
**Leucine, μmol/L**										
Day 70	210.5	220.3	202.8	10.62	0.82	0.74	0.66	0.66	0.02	0.84
Day 90	217.6	228.4	205.7	6.21	0.36	0.35	0.26			
Day 110	194.8	165.5	168.0	13.38	0.61	0.48	0.54			
**Lysine, μmol/L**										
Day 70	281.5	321.0	239.5	18.85	0.17	0.24	0.19	0.44	<0.01	0.71
Day 90	409.4	423.6	438.9	18.45	0.83	0.55	0.93			
Day 110	282.4	326.5	296.3	14.23	0.48	0.85	0.25			
**Methionine, μmol/L**										
Day 70	48.5	57.7	50.8	2.80	0.47	0.92	0.24	0.48	<0.01	0.92
Day 90	73.0	72.4	68.6	2.48	0.76	0.47	0.86			
Day 110	58.6	62.3	59.0	2.20	0.79	0.96	0.50			
**Ornithine, μmol/L**										
Day 70	208.0	163.3	146.0	17.04	0.43	0.22	0.58	0.12	<0.01	<0.01
Day 90	149.6 ^b^	194.1 ^a^	228.1 ^a^	9.85	<0.01	<0.01	0.32			
Day 110	114.4^b^	143.8^b^	184.6^a^	9.38	<0.01	<0.01	0.80			
**Phenylalanine, μmol/L**										
Day 70	93.0	98.0	84.5	13.60	0.53	0.42	0.52	0.24	0.07	0.77
Day 90	85.5	104.8	85.6	4.51	0.13	0.75	0.05			
Day 110	79.3	81.7	76.4	3.83	0.89	0.72	0.71			
**Proline, μmol/L**										
Day 70	408.0	422.0	379.5	21.51	0.73	0.59	0.67	0.75	0.21	0.75
Day 90	389.5	400.6	434.3	13.71	0.42	0.19	0.87			
Day 110	349.8	386.5	377.7	15.44	0.60	0.55	0.47			
**Serine, μmol/L**										
Day 70	186.5	179.7	153.8	7.66	0.17	0.09	0.75	0.26	0.08	0.83
Day 90	167.6	215.6	158.0	16.59	0.33	0.64	0.16			
Day 110	139.5	153.3	134.6	7.01	0.61	0.65	0.35			
**Taurine, μmol/L**										
Day 70	141.5	119.0	83.0	13.35	0.22	0.10	0.96	0.13	<0.01	0.14
Day 90	83.0	92.9	88.4	4.40	0.67	0.72	0.42			
Day 110	67.8	66.2	72.9	2.96	0.68	0.44	0.62			
**Threonine, μmol/L**										
Day 70	177.0	191.7	161.3	13.47	0.68	0.61	0.56	0.05	0.05	0.82
Day 90	138.1	149.1	126.0	6.86	0.42	0.39	0.31			
Day 110	133.2	167.2	105.6	11.06	0.11	0.16	0.08			
**Tryptophan, μmol/L**										
Day 70	61.0	76.0	62.5	4.37	0.36	0.92	0.19	0.10	0.40	0.85
Day 90	62.8	67.1	64.1	2.35	0.75	0.91	0.46			
Day 110	54.6	66.5	60.0	3.00	0.29	0.62	0.15			
**Tyrosine, μmol/L**										
Day 70	98.0	104.7	91.5	7.10	0.77	0.69	0.63	0.17	0.23	0.96
Day 90	98.1	120.9	97.7	6.37	0.24	0.77	0.10			
Day 110	87.1	101.3	89.7	4.03	0.36	0.99	0.16			
**Valine, μmol/L**										
Day 70	306.5	330.3	284.0	15.04	0.46	0.48	0.39	0.25	0.01	0.93
Day 90	276.9 ^AB^	293.6 ^A^	253.4 ^B^	7.49	0.09	0.12	0.10			
Day 110	252.0	230.3	202.7	19.14	0.57	0.31	0.93			

^1^ CON: corn-SBM-based diet with Arg 0.72%, Arg10: corn-SBM-based diet with Arg 1.0%, Arg15: corn-SBM-based diet with Arg1.5%. ^2^ Standard error of the mean. ^a,b^ Means in the same row with different superscript letters were significantly different (*p* < 0.05). ^A,B^ Means in the same row with different superscript letters were significantly different (*p* < 0.10). Lin.: linear, Quad.: quadratic.

**Table 7:** Effects of arginine supplementation levels on blood profiles in late-gestating sows.

	Treatment ^1^			SEM ^2^	*p*-Value					
	CON	ARG10	ARG15		Diet	Lin.	Quad.	Group	Date	Group × Date
**Blood urea nitrogen, mg/dL**										
Day 70	12.5	11.7	11.9	0.45	0.85	0.72	0.63	0.99	0.01	0.93
Day 90	11.3	12.0	12.5	0.57	0.69	0.41	0.83			
Day 110	14.3	14.5	14.1	0.57	0.56	0.84	0.83			
**Creatinine, mg/dL**										
Day 70	1.76	1.74	1.95	0.091	0.59	0.41	0.70	0.48	<0.01	0.89
Day 90	2.23	2.43	2.39	0.104	0.74	0.63	0.55			
Day 110	2.97	2.90	3.18	0.097	0.48	0.32	0.50			
**Total protein, g/dL**										
Day 70	6.85	6.67	7.15	0.256	0.76	0.63	0.67	0.95	0.98	0.65
Day 90	6.86	7.23	6.71	0.170	0.47	0.60	0.27			
Day 110	6.93	6.73	7.00	0.175	0.82	0.80	0.57			
**Urea, mg/dL**										
Day 70	26.8	25.0	25.5	0.97	0.85	0.73	0.62	0.99	0.01	0.93
Day 90	24.1	25.6	26.7	1.22	0.69	0.42	0.83			
Day 110	30.6	31.1	30.1	1.22	0.96	0.84	0.83			

^1^ CON: corn-SBM-based diet with Arg 0.72%, Arg10: corn-SBM-based diet with Arg 1.0%, Arg15: corn-SBM-based diet with Arg1.5%. ^2^ Standard error of the mean. Lin.: linear, Quad.: quadratic.

**Table 8:** Effects of arginine supplementation levels during late gestation on milk composition in lactating sows.

	Treatment ^1^			SEM ^2^	*p*-Value		
	CON	ARG10	ARG15		Diet	Lin.	Quad.
**Casein, %**							
Colostrum	6.57	6.08	5.62	0.571	0.81	0.52	0.91
Milk (21 days)	4.05	4.32	4.09	0.119	0.63	0.98	0.34
**Fat, %**							
Colostrum	6.68	5.43	6.91	0.485	0.43	0.69	0.23
Milk (21 days)	4.04	6.82	6.32	0.368	0.13	0.23	0.10
**Protein** **, %**							
Colostrum	8.28	7.91	7.29	0.804	0.89	0.63	0.99
Milk (21 days)	4.72	4.77	4.61	0.115	0.86	0.68	0.72
**Lactose, %**							
Colostrum	4.72	4.35	4.31	0.187	0.65	0.44	0.60
Milk (21 days)	5.77	6.26	5.91	0.134	0.32	0.86	0.14
**Total Solid** **, %**							
Colostrum	21.9	20.1	20.9	0.76	0.67	0.67	0.43
Milk (21 days)	16.9	19.2	18.1	0.54	0.23	0.49	0.12
**Solid not Fat, %**							
Colostrum	13.3	12.7	11.9	0.68	0.74	0.44	0.93
Milk (21 days)	10.7	11.0	10.6	0.17	0.64	0.66	0.41

^1^ CON: corn-SBM-based diet with Arg 0.72%, Arg10: corn-SBM-based diet with Arg 1.0%, Arg15: corn-SBM-based diet with Arg1.5%. ^2^ Standard error of the mean. Lin.: linear, Quad.: quadratic.
